# Multi-Reader–Multi-Split Annotation of Emphysema in Computed Tomography

**DOI:** 10.1007/s10278-020-00378-2

**Published:** 2020-08-10

**Authors:** Mats Lidén, Ola Hjelmgren, Jenny Vikgren, Per Thunberg

**Affiliations:** 1 Department of Radiology, Faculty of Medicine and Health, Örebro University, Örebro, Sweden; 2 Department of Molecular and Clinical Medicine, Institute of Medicine, Sahlgrenska Academy, University of Gothenburg, Gothenburg, Sweden; 3 Department of Clinical Physiology, Region Västra Götaland, Sahlgrenska University Hospital, Gothenburg, Sweden; 4 Department of Radiology, Sahlgrenska University Hospital and Institute of Clinical Sciences, Sahlgrenska Academy, University of Gothenburg, Gothenburg, Sweden; 5 Department of Medical Physics, Faculty of Medicine and Health, Örebro University, Örebro, Sweden

**Keywords:** Computed Tomography, X-Ray, Chronic Obstructive Pulmonary Disease, Pulmonary Emphysema, Machine Learning, Image Annotation, Observer Variation

## Abstract

Emphysema is visible on computed tomography (CT) as low-density lesions representing the destruction of the pulmonary alveoli. To train a machine learning model on the emphysema extent in CT images, labeled image data is needed. The provision of these labels requires trained readers, who are a limited resource. The purpose of the study was to test the reading time, inter-observer reliability and validity of the multi-reader–multi-split method for acquiring CT image labels from radiologists. The approximately 500 slices of each stack of lung CT images were split into 1-cm chunks, with 17 thin axial slices per chunk. The chunks were randomly distributed to 26 readers, radiologists and radiology residents. Each chunk was given a quick score concerning emphysema type and severity in the left and right lung separately. A cohort of 102 subjects, with varying degrees of visible emphysema in the lung CT images, was selected from the SCAPIS pilot, performed in 2012 in Gothenburg, Sweden. In total, the readers created 9050 labels for 2881 chunks. Image labels were compared with regional annotations already provided at the SCAPIS pilot inclusion. The median reading time per chunk was 15 s. The inter-observer Krippendorff’s alpha was 0.40 and 0.53 for emphysema type and score, respectively, and higher in the apical part than in the basal part of the lungs. The multi-split emphysema scores were generally consistent with regional annotations. In conclusion, the multi-reader–multi-split method provided reasonably valid image labels, with an estimation of the inter-observer reliability.

**Electronic supplementary material:**

The online version of this article (10.1007/s10278-020-00378-2) contains supplementary material, which is available to authorized users.

## Introduction

Chronic obstructive pulmonary disease (COPD) is a major respiratory health problem, mainly caused by cigarette smoking, with high morbidity and mortality. In 2002, COPD was the fifth leading cause of death worldwide [1]. COPD is characterized by a variable combination of small airway disease and emphysema (destruction of the alveoli) [2].

With the variable combination of emphysema and airway involvement, the same severity of COPD may manifest as different patterns on computed tomography (CT) images. Pulmonary function test (PFT) is the standard for diagnosis, while CT is a complementary method offering additional information [2, 3].

By giving the volume of the low attenuating area in the lungs, quantitative CT (qCT) has shown an important correlation to the severity of COPD, and is an independent predictor of morbidity and mortality in COPD patients [4–6]. However, counting low-density pixels does not gather all image information, since visual emphysema scoring is an independent predictor even in models where qCT is included, and qCT may show similar results in patients with and without visual emphysema [6, 7]. Machine learning models can learn image features from training data, and there are expectations of improved quantitative emphysema scoring [8].

Acquiring annotations for image data is a time-consuming step in the machine learning pipeline, and is often a bottleneck in radiological applications [9–11]. In medical imaging, the labels need to be provided by qualified readers and, depending on the task, at a sufficiently detailed level. For COPD applications, labels may be provided at an overall level, for example using PFT as a reference test or an overall visual emphysema score, or may be provided in more detail, with pixel-wise emphysema segmentation or a detailed emphysema score.

Overall labels provide a coarse annotation, but pixel-wise segmentation may not be feasible because of the large effort required, as well as low repeatability [12]. A midway approach is to obtain visual scores for each slice in the CT scan, without the need for classification of each pixel. Slice-wise scores can be combined into an aggregated score for each patient, for use in correlation with clinical outcomes.

Detailed image labels are commonly provided by one or several readers [13–15]. Obtaining visual emphysema scores from only a few readers has some disadvantages, such as that the generalizability of the annotations and the estimate of inter-observer reliability may be questioned, and the labeling may be too much work for a single reader.

A different approach is to split the annotation into a large number of independent subtasks that are distributed to multiple readers. In the multi-reader–multi-split method, we divide the annotation of each CT scan into many independent subtasks that are completed by different readers. In many fields, researchers depend on observational data obtained by observers. The multi-reader–multi-split method, where the assessment of each subject is divided into multiple subtasks, is an example of not fully crossed design where multiple readers rate a subset of tasks [16].

To the best of our knowledge, there is no previous report in CT imaging on trying a method for acquiring image labels, where different slices in a single CT scan are labeled by different readers. With the overall aim of testing the multi-reader–multi-split method and its inter-observer reliability for annotating CT image data, the objectives of the present study were to evaluate (1) the duration of the reading sessions; (2) the inter-observer reliability in emphysema classification and scoring; and (3) the validity of the annotations by comparing the multi-reader–multi-split annotations with conventional regional annotations.

## Material and Methods

### SCAPIS Pilot Image Data and Annotations

The Swedish CArdioPulmonary bioImage Study (SCAPIS) is a national, multi-center, cross-sectional cohort study including cardiac and thoracic CT scans, vascular ultrasound, blood samples, and functional tests of more than 30,000 individuals aged 50–64. The aim of SCAPIS is to predict and prevent cardiovascular disease and COPD [17].

Preceding SCAPIS, the SCAPIS pilot included 1111 participants in Gothenburg, Sweden, between February and November 2012 [17]. At inclusion in the SCAPIS pilot, the emphysema extent in the CT images was assessed for each patient and registered in an electronic case report form (eCRF). The emphysema extent was annotated in three separate regions in each lung, at each examination: upper lung (between apex and carina), middle lung (from carina to the lower pulmonary vein), and lower lung (between the pulmonary vein and the lung base). The emphysema type was determined using the classifications “none,” “centrilobular,” “paraseptal,” “combined centrilobular and paraseptal,” “panlobular,” and “bullae.” For any emphysema type, the degree was assessed as mild (1–25%), moderate (> 25–50%), or severe (> 50%). These regional annotations were obtained prior to the present study, by experienced senior thoracic radiologists, and were used for comparison with the multi-reader–multi-split annotations obtained in this study [7].

The study protocol was approved by the regional research ethics board. Informed consent was signed at inclusion in the SCAPIS pilot.

### Image Data and Chunk Preparation

From the SCAPIS pilot, thoracic CT scans from a subset of 100 subjects with visible emphysema, and 100 matched controls without emphysema, have previously been selected [7]. The images in the present study were extracted from these scans. In total, CT images of 102 subjects were included in the study, 96 with emphysema and six controls. In four subjects in the pre-selected emphysema cohort, thin-slice CT images were not available; these patients were excluded. The severity of emphysema in most patients in the emphysema cohort was mild, with many regions without emphysema according to the eCRF [7]. To avoid worsening the skewed distribution of regional emphysema scores, only six randomly selected subjects from the matched controls were included in the present study.

The CT images were acquired on a Siemens Somatom Definition Flash (Siemens, Erlangen, Germany), reference tube voltage and reference mAs for CARE dose 4D, 120 kVp and 25 mAs, respectively. The median effective dose was 2 mSv. Images were reconstructed as 0.6-mm contiguous slices with a soft tissue algorithm (B31 (*n* = 42), I31f2 (*n* = 12), or I31f3 (*n* = 48)).

From each CT stack, all slices between the pulmonary apex and base were extracted using a software developed for the study. Each examination was split into 1-cm chunks (17 slices of 0.6 mm, per chunk). The mean number of chunks per examination was 28 (range 23–33), giving 2881 separate chunks in total.

**Fig. 1:**
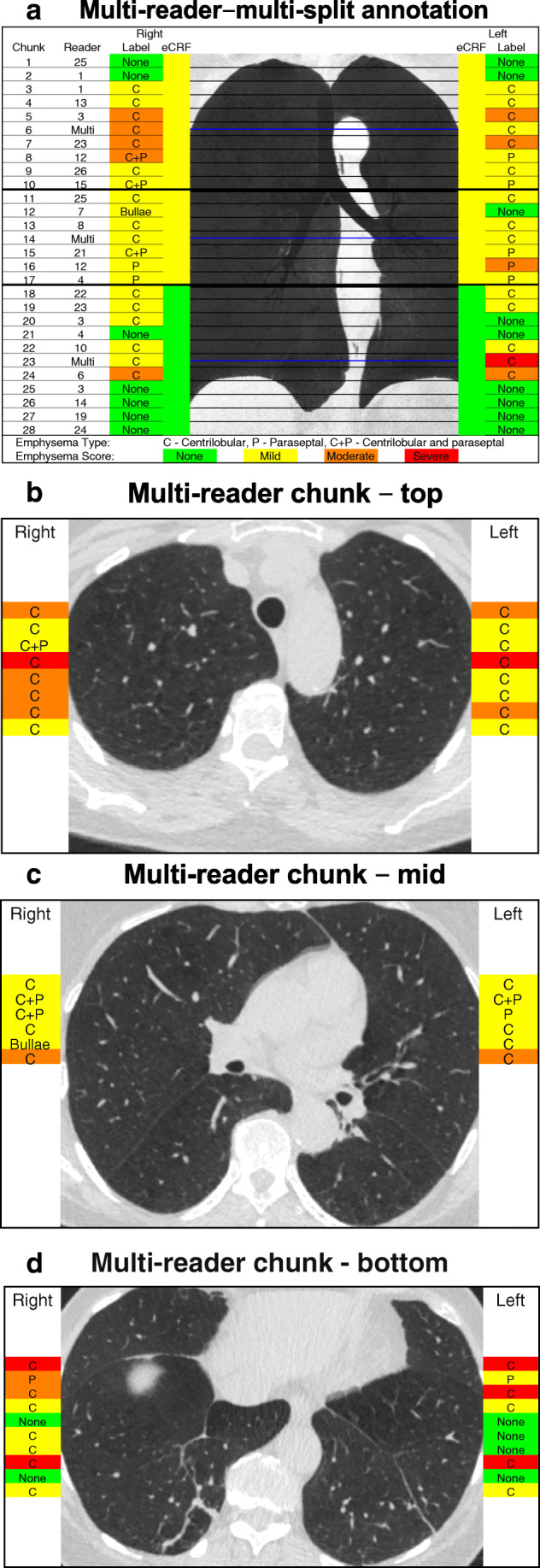
Multi-reader–multi-split annotation in one subject. The overall electronic case report form (eCRF) emphysema type was centrilobular. The multi-split emphysema score is color-coded and type is abbreviated. (**a**) Coronal minimum intensity (MinIP) projection demonstrating the 28 chunks from this subject. The multi-reader chunks are indicated by blue lines. The regional eCRF score is color-coded. The reader variations in the top, middle, and bottom multi-reader chunks are shown in Fig. 1**b**–**d**. The number of readers in the multi-reader chunks varies because of the random sampling. The MinIP images were not available for the readers

Three chunks per examination formed the *multi-reader chunks*. These were located in the middle of each of the upper, middle, and lower lung zone (see Fig. 1). These 306 multi-reader chunks were annotated by up to 13 readers (median six). The remaining 2575 *single-reader chunks* were annotated by one reader each.

### Image Review

Twenty-six readers were included in the study, comprising radiologists and radiology residents. The task for each reader was to assess the type and degree of emphysema in the left and right lung in 175 CT chunks; 75 chunks were randomly selected from the *multi-reader chunks*, and 100 chunks were randomly selected from the *single-reader chunks*.

For each chunk, the reader provided two assessments—for the right and left lung parenchyma separately. The emphysema type and degree was assessed using the same classification as used for the regional assessment at inclusion in the SCAPIS pilot described above. The readers received a written instruction including images with examples of all emphysema scores for all emphysema subtypes, and a short oral instruction.

The assessment of each chunk was performed in an application where the readers could scroll through the 17 axial slices similar to a standard radiological workstation (see supplementary material). The CT slices were displayed in lung window setting (C-500/W 1500) and were zoomed × 1.5 from pixel-to-pixel size. The application was custom-built for the study in Matlab R2018b (The Mathworks, Natick, MA, USA).

### Reading Time Analysis

For each annotated chunk, the reading time was recorded. The per chunk reading time for chunks with and chunks without emphysema was compared using Wilcoxon’s rank sum test. Reading times > 3 min/chunk were considered pauses and excluded from the reading time analysis.

### Inter-observer Reliability—Mutli-Reader Chunks

#### Krippendorff’s Alpha

The inter-observer reliability in the emphysema type and score for the multi-reader chunks was analyzed using Krippendorff’s alpha, where alpha = 0 represents no agreement and alpha = 1 represents perfect agreement [18–20]. In contrast to the more common Cohen’s kappa, Krippendorff’s alpha can handle any number of readers, missing values and data on both nominal and numerical scales. A rule for interpreting Krippendorff’s alpha for a specific context is difficult to find. For reliability data, alpha > 0.66 is sometimes considered acceptable, but requires interpretation depending on context [18].

Confidence intervals (CIs) were obtained through bootstrapping with 1000 replicates using the K_alpha script provided by Zapf et al. [19].

#### Systematic Differences Between Readers

The tendency for each reader to assign a certain type and score was analyzed by counting the numbers of scores assigned to each class for each reader in the multi-reader chunks. The distribution of eCRF regional scores in the chunks for each reader was also analyzed to assess whether the random assignments of chunks were homogeneous between the readers.

The differences in emphysema type and score, and the distribution of eCRF regional scores between readers were tested for homogeneity with chi squared tests, with the null hypothesis of equal distributions independent of reader.

### Consistency Between Adjacent Chunks

Emphysema scores for adjacent chunks are expected to show a smooth variation in the cranio-caudal direction due to the diffuse nature of emphysema. The consistency in emphysema score between adjacent chunks was analyzed on an ordinal scale by counting the difference between the emphysema score provided in a chunk and the adjacent chunks.

The difference between severe emphysema and no emphysema received absolute difference 3; the difference between severe and mild emphysema and between moderate and no emphysema received difference 2; and the difference between severe and moderate emphysema, between moderate and mild emphysema and between mild and no emphysema, received difference 1.

### Validity of Annotations—per Chunk vs. Regional Annotation

The per chunk emphysema scores were compared with the eCRF regional emphysema score assigned at inclusion in SCAPIS pilot in the corresponding region [7]. For each eCRF emphysema score (no emphysema, mild, moderate, and severe emphysema), the number and proportion of corresponding per chunk emphysema scores were computed. The proportion of per chunk emphysema scores for the eCRF regional scores were tested for homogeneity using chi squared test with the null hypothesis of equal distributions independent of eCRF score.

### Statistics

Matlab was used for statistics, except for computation of Krippendorff’s alpha CIs, where an R (R Foundation for Statistical Computing, Vienna, Austria) script (K_alpha) was used [19].

## Results

### Baseline Characteristics

Baseline characteristics of included patients are given in Table 1.

**Table 1:** Baseline characteristics of included subjects

Background data	
Participants, *n* (male/female)	102 (55/47)
Age (years)	58 ± 5
Body weight (kg)	76 ± 16
Height (m)	1.70 ± 0.1
BMI (kg/m^2^)	26 ± 5

Values are given as mean ± standard deviation. *BMI* body mass index

Between May and September 2019, altogether, 26 readers provided a total of 9050 separate assessments of emphysema type and severity in 2881 separate chunks from 102 participants. The number of assessments exceeded the number of chunks because the left and right lungs were separately rated, and several readers rated the same multi-reader chunks. Fifteen of the participating readers were radiologists, with a median of 8 (range 0–33) years’ experience after certification. Eleven readers were radiology residents, median third year (range 0–6 years).

### Reading Time

The median number of reading sessions for annotating the 175 chunks was two (range one to four). The median reading time per chunk was 15 s (interquartile range 17 s). The median reading time was longer for chunks with emphysema compared with that of chunks assessed as normal (*p* < 0.001), 20 s vs. 10 s.

### Inter-observer Reliability

### Multi-Reader Chunks—Emphysema Type

The overall Krippendorff’s alpha for the nominal-scaled emphysema type was 0.40 (95% CI 0.37–0.43). The agreement was higher in the upper than that in the lower part of the lungs. For multi-reader chunks in the upper, middle, and lower part of the lung, alpha (95% CI was) was 0.45 (0.40–0.49), 0.31 (0.26–0.35), and 0.29 (0.23–0.35), respectively.

The confidence intervals for the upper part of the lungs did not overlap with the mid or lower part, indicating significant difference at *p* < 0.05 level.

### Multi-Reader Chunks—Emphysema Score

The overall Krippendorff’s alpha, for the ordinal-scaled emphysema score (0–3), was 0.53 (95% CI 0.49–0.57). Similar to the assessment of emphysema type, the agreement was higher in the upper than that in the lower part of the lungs. For multi-reader chunks in the upper, middle, and lower part of the lung, alpha (95% CI) was 0.59 (0.53–0.65), 0.45 (0.37–0.52), and 0.39 (0.30–0.47), respectively.

The confidence intervals for the upper part of the lungs did not overlap with the mid or lower part, indicating significant difference at *p* < 0.05 level.

### Systematic Differences Between Readers

**Fig. 2:**
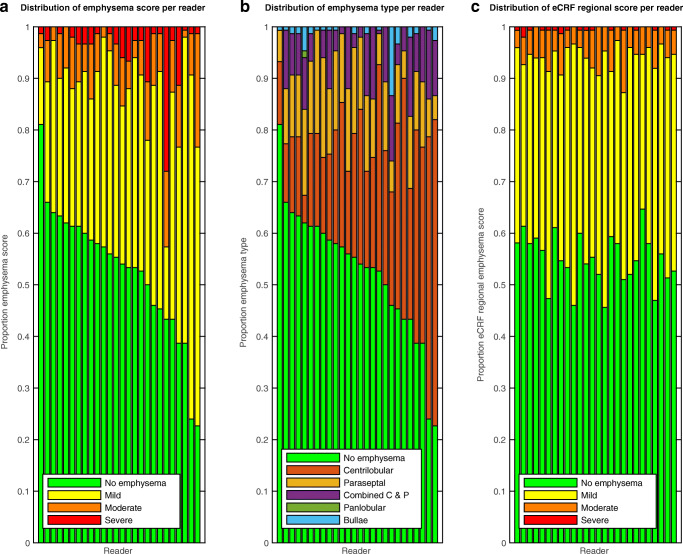
Systematic differences between readers in (**a**). emphysema score and (**b**). emphysema type, and (**c**). Absence of systematic difference in corresponding eCRF score. Each bar represents a reader. The colors represent the relative frequencies of the classifications for the reader. The 26 readers are sorted according to the proportion of normal lung parenchymas, with maintained positions in (**a**), (**b**) and (**c**). Lung parenchyma in both sides in multi-reader chunks are included

There was a systematic difference between readers in the assessment of emphysema type and emphysema score, both *p* < 0.001 according to chi squared test. Some readers tended to report lower emphysema scores, while others reported higher scores. Figure 2 a and b shows the systematic differences between readers in the assessment of emphysema score and emphysema type. There was no systematic difference in eCRF score in the random assignment of the multi-reader chunks, *p* = 0.09 (see Fig. 2c).

### Consistency Between Adjacent Chunks

**Fig. 3:**
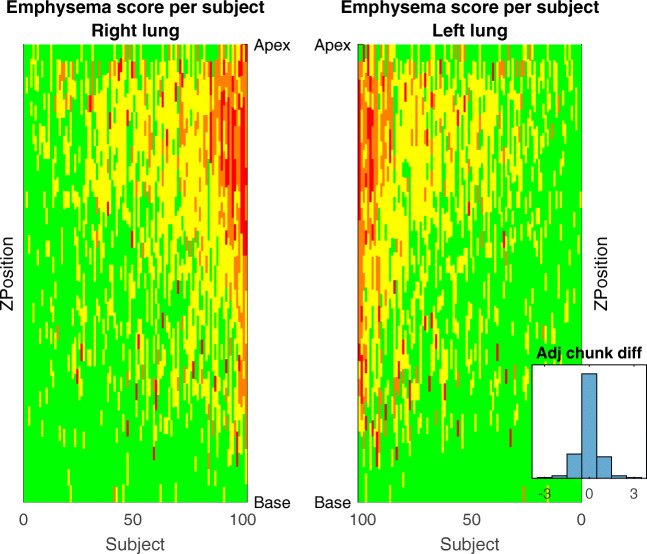
Multi-reader–multi-split emphysema scores for both lungs for all subjects in the study. The emphysema degree is color-coded; green, yellow, orange, and red represent no, mild, moderate and severe emphysema, respectively. The subjects are sorted according to mean total emphysema score. A vertical line for each lung demonstrates the cranio-caudal distribution of emphysema scores for each subject. The histogram of the difference between adjacent chunks is inserted

Figure 3 shows the emphysema score in all chunks for the right and left lungs, sorted according to the total emphysema score for the patient.

The median absolute difference between the scores on adjacent chunks was zero, demonstrating that, in the majority of assessments, the score was identical for adjacent chunks. A visual assessment of Fig. 3 reveals the expected, predominantly apical distribution of emphysema and a general consistency between adjacent chunks.

There are, however, several distinctive annotations that clearly differ from adjacent annotations, for example, for single chunks graded as severe emphysema among chunks graded as normal. The identification of outlying annotations indicates where additional measures to reduce variation can be directed. However, this step is beyond the scope of the present manuscript.

### Validity of Annotations—per Chunk vs. Regional Annotation

For multi-reader chunks, the median score of the most common emphysema type was used. Most subjects from the emphysema cohort had several segments without emphysema, according to the eCRF classification. In addition, the six subjects from the control group had no segments with emphysema. Consequently, the most common emphysema score, as given on the eCRF, was no emphysema (327 regions), followed by mild, moderate, and severe emphysema (in 245, 35, and three regions, respectively). The number of multi-split assessments for no emphysema, as per the eCRF, was 3119, compared with 2253 for mild, 344 for moderate, and 28 for severe emphysema. Since the eCRF score was missing for two regions, the number of comparisons using the eCRF is lower than twice the number of chunks in the study.

**Fig. 4:**
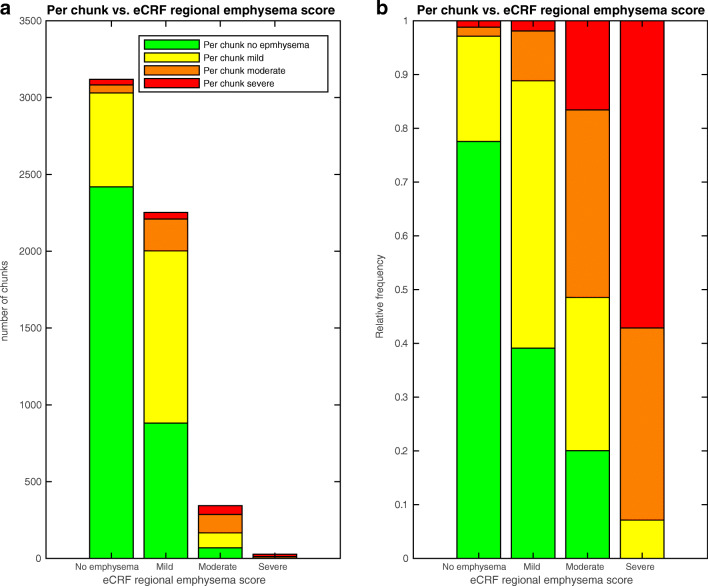
(**a**) Absolute and (**b**) relative distribution of emphysema score for chunks within different eCRF regional emphysema score

The multi-reader–multi-split labels were related to the eCRF scores according to chi squared test, (*p* < 0.001) (see Fig. 4); for example, in regions that had the eCRF classification “mild emphysema,” 50% of multi-split labels indicated mild emphysema and 39% said no emphysema. Considering the expected cranio-caudal distribution of emphysema, as shown in Fig. 3, many multi-split assessments in regions with mild emphysema are expected to be normal. The concordance with the eCRF indicates that the multi-reader–multi-split annotations measured the same entity as the regional emphysema scores, and therefore suggests that the labels are valid.

## Discussion

In the present study, we investigated the feasibility of the multi-reader–multi-split method for annotating emphysema in pulmonary CT. The coherence to the eCRF annotations indicated that the multi-split annotations were valid. The inter-observer reliability analysis, based on a large number of included readers, revealed a variability with a cranio-caudal gradient within the lungs. The inter-observer reliability may serve as a benchmark for future machine learning approaches.

Several aspects need to be analyzed here, considering the feasibility of the method for acquiring annotations. Among these aspects are how to recruit readers, how to make the annotation session tolerable for the readers, and the generalizability, validity, and precision of the labels.

Labeled images are needed for the development of machine learning methods. In medical applications, they need to be provided by qualified readers, who are a limited resource [9, 10]. Compared with normal clinical work, image labeling may also be a tedious task, which may hamper the recruitment of readers. In the present study, we overcame these limitations by introducing the multi-reader–multi-split method. The large workload of providing detailed emphysema scores was split into more than 9000 separate assessments randomly distributed to 26 readers, where each label required only a few seconds.

The multi-reader–multi-split method is, to the best of our knowledge, a new application in radiology of a method where different parts of the same CT examination is labeled by different readers. Besides lowering the workload for each reader, the multi-reader–multi-split method also enabled the analysis of the inter-observer reliability.

A machine learning method aimed to add information to the PFT and qCT in predicting the clinical outcome in COPD patients is the next phase of the project, but this is beyond the scope of the present study. The absence of difference in qCT values between cases and controls in the same cohort emphasizes the need for an automated method that coheres to the visual scores [7].

Although more detailed annotation levels capture more information, pixel-wise annotations in diffuse pulmonary disease are generally not achievable because there is no distinct border between healthy and unhealthy lung tissue [12]. In the present study, we instead used a visual emphysema score obtained for each centimeter on the *z*-axis of the thoracic CT image. These can be aggregated into a more detailed score for the patient than the regional eCRF scores.

Labels provided by expert readers are often used as ground truth [10]. The systematic differences between readers, as demonstrated by Fig. 2, emphasize the importance in the selection of the reader if a single reader labels images. In contrast, with the multi-reader method, the impact of each reader is smaller.

In the present study, we analyzed the validity by comparing the multi-reader annotations to the independent eCRF annotations [7]. Figure 4 shows that 98% of the chunks in regions assessed as normal using the eCRF were labeled as no or mild emphysema, and 93% of the chunks in regions assessed as severe emphysema in the eCRF were labeled as moderate or severe emphysema. Complete agreement between the multi-reader–multi-split labels and the eCRF for the diffusely distributed lung disease is not desirable, since the eCRF annotations are regional—each region approximately 9 cm—while the multi-split annotations are created for each centimeter. Instead, the reasonable agreement found in the present study indicates that the annotations are reasonably valid, and that the slice-wise labels may be combined into a more detailed score compared with that of the regional eCRF scores.

The precision of the labels refers to the repeatability. By acquiring labels from multiple readers, the inter-observer reliability has been quantified. The inter-observer reliability requires an interpretation beyond a single statistic measure. Although Krippendorff’s alpha in the present study is generally below what is considered acceptable repeatability, the reason for inter-observer variations must be taken into account.

In the present study, Krippendorff’s alpha concerning emphysema score was 0.39, 0.45, and 0.59 on the multi-reader chunks in the lower, mid, and upper parts of the lungs, respectively. In a previous study concerning the eCRF regional scores in the same cohort, the corresponding inter-observer alpha for two included readers was between 0.51 and 0.76, while the intra-observer alpha for three readers was between -0.07 and 0.90 [7].

A lower agreement in the present study was anticipated since the readers only had access to very limited data, 1 cm per chunk. Importantly, the wide range of Krippendorff’s alpha in the present study and in the previous study illustrates that complete agreement between visual emphysema score is not achievable. Complete agreement is only possible if there is an objective correct emphysema score, but visual scoring is subjective and the cutoff points are arbitrary. The distribution of scores from many readers in the present study illustrates that rather than estimating the correct score for a given chunk, a given score should be seen as an observation of the distribution of possible scores from qualified readers for the chunk (see Fig. 1, 2, and 3). With several readers, this distribution can be estimated.

For use in machine learning, a discrete classification of emphysema, rather than a distribution, for a given chunk is necessary, but the discrete classes rely on a simplification of the radiological interpretation. To interpret the outcome of a machine learning algorithm, the estimation of the inter-observer reliability between radiologists is therefore necessary.

Consistent with previous findings, the inter-observer variations were largest in the basal part of the lungs [7]. Multiple factors may have contributed to this finding. In the basal part of the lung, there are more motion artifacts. In the apical part of the lungs, the emphysema may have a more typical appearance, and the cross-sectional area of the lung is smaller.

This study has several limitations. For efficient sequential viewing of multiple chunks from different examinations, a custom graphical user interface was required. Although the viewer was designed to resemble a standard radiological workstation, its functionality was limited. The limited number of 17 axial slices per chunk, without multiplanar reformats, may not be ideal for evaluation. Although the number of chunks was large, all chunks originated from 102 subjects. A larger patient cohort may be necessary for improved generalizability. The intra-observer reliability could not be assessed.

The study provided a visual method for identifying outlying annotations. Before starting the machine learning process, improvement of the data including outlying chunk labels may be necessary. A further development would also be to create a web-based annotation tool, which would provide more convenient access for the readers, and enable recruitment of readers from multiple sites.

## Conclusions

The present study indicates that the multi-reader–multi-split method for acquiring medical image is tolerable for the readers, leads to reasonably valid image labels, and enables an important analysis of the inter-observer reliability.

## Electronic Supplementary Material

Fig. 1 The annotation application, where the reader could scroll through the 17 axial slices of each chunk. (PNG 803 kb)

High Resolution (TIF 541 kb)
