# Supplementary figures and images for: Multi-Reader–Multi-Split Annotation of Emphysema in Computed Tomography
Source: J Digit Imaging. 2020 Aug 10;33(5):1185–93. doi: 10.1007/s10278-020-00378-2 (PMC7572947; doi:10.1007/s10278-020-00378-2)

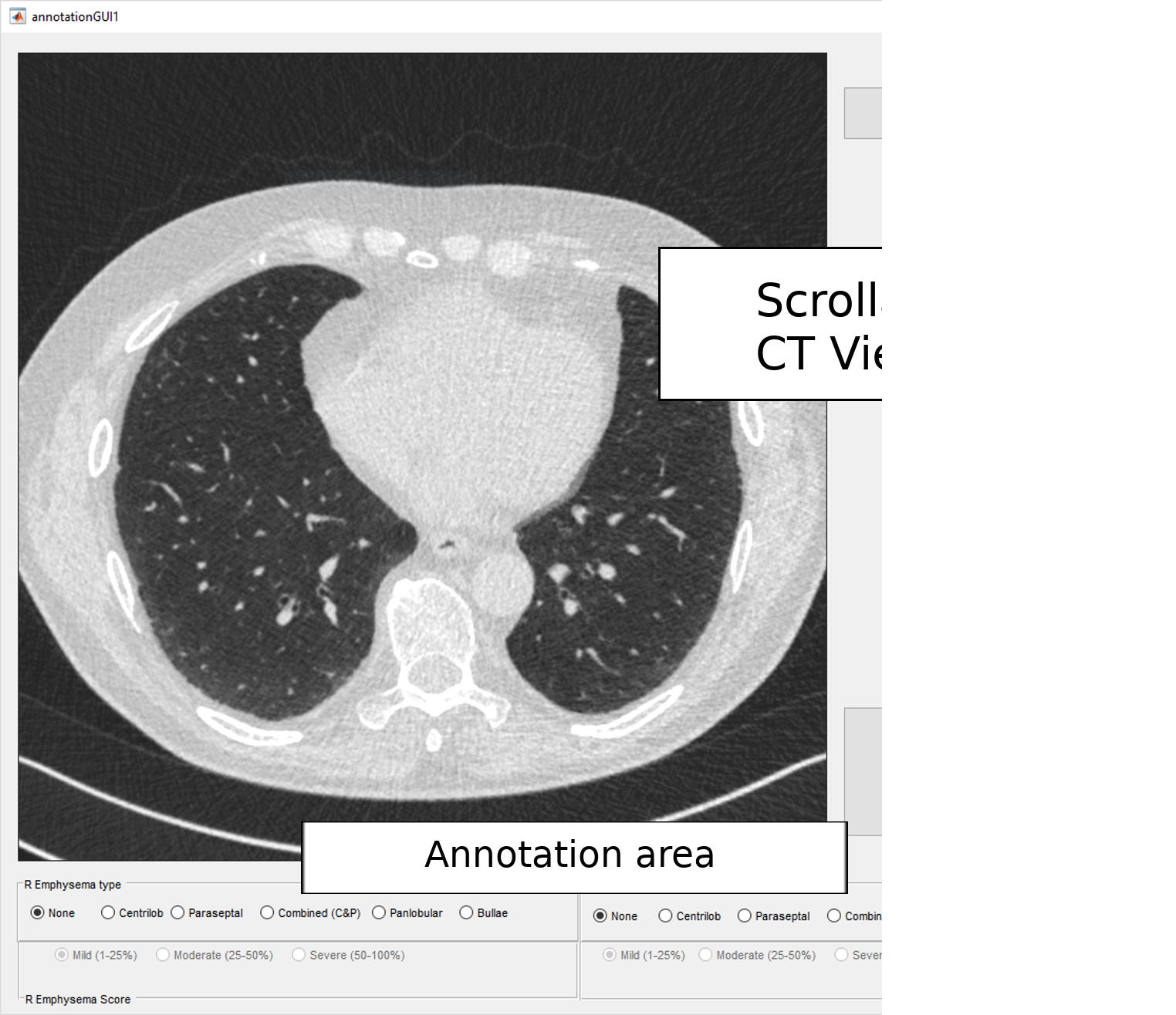

Supplement: Supplementary file 1 — The annotation application, where the reader could scroll through the 17 axial slices of each chunk. (PNG 803 kb) [file 10278_2020_378_Fig5_ESM.png]
